# Ethical Challenges of Germline Genetic Enhancement

**DOI:** 10.3389/fgene.2019.00767

**Published:** 2019-09-03

**Authors:** Ignacio Macpherson, María Victoria Roqué, Ignacio Segarra

**Affiliations:** ^1^Department of Humanities, International University of Catalonia, Barcelona, Spain; ^2^Pharmacokinetics, Patient Care and Translational Bioethics Research Group, Catholic University of Murcia (UCAM), Murcia, Spain; ^3^Department of Pharmacy, Faculty of Health Sciences, Catholic University of Murcia (UCAM), Murcia, Spain

**Keywords:** genetic enhancement, human identity, genetic interventions, reprogenetic, human procreation, precautionary principle, ELSI

## Abstract

The new reproductive technologies have opened the door to different processes of germline genetic enhancement by which the characteristics of an individual according to the interests of the agents involved could be selected during its gestation. Although the initiative is apparently oriented towards developing individuals that would excel in society, critical voices raise the concerns about that this approach would generate and need for a reflection on the ethical, social and legal implications of these techniques and their implementation in society. We reviewed the literature about these issues throughout their historical records to date, focusing on the moral arguments and non-clinical aspects that affect the legal and social environment. We have observed various trends of thought with divergent positions (proactive, preventive, and regulatory) as well as a large number of articles that try to reconcile the different approaches. This review illustrates a series of concepts from the ethics and philosophy fields which are frequently used in studies that evaluate the ethical implications of germline genetic enhancement, such as dignity, benefit, autonomy, and identity. In addition, amongst the many unresolved controversies surrounding genetic enhancement, we identify procreative beneficence, genetic disassociation, gender selection, the value of disability, embryo chimerization, and the psychosocial inequality of potentially enhanced individuals as crucial. We also develop possible scenarios for future debate. We consider especially important the definition and specification of three aspects which are essential for the deployment of new reproductive technologies: the moral status of the embryo undergoing enhancement, the legal status of the enhanced individual, and the responsibility of the agents executing the enhancement. Finally, we propose the precautionary principle as a means to navigate ethical uncertainties.

## Introduction

The interest for the physical, moral, or cognitive well-being of all human individuals and its transmission to following generations has existed in the minds of the wise and rulers for thousands of years. Accomplishing this objective demands to specify the means to reach it, something especially difficult when it comes to transmitting the biological features of parents to children, that is, the genetic inheritance. Throughout history, civilizations have implemented various means to achieve the correct transmission of biological inheritance, generally through negative laws that forbid the pairing of consanguineous individuals as well as incest (Bittles, 2003). In these cases, the laws were not intended to “enhance” the inherited characteristics rather to avoid the emergence of diseases or inbred disabilities (Güvercin and Arda, 2008), and the “worsening” of the offspring. However, in Plato’s Republic we may find the formulation of one of the first political theories trying to enhance the individuals of a society with proactive means. Amongst Plato’s proposals we may find the selection of couples, the controlled inbreeding and crossing, the classification of newborns and their upbringing, or abandonment according to their physiological characteristics (Güvercin and Arda, 2008). The genetic enhancement proposal appeared again strongly at the end of the 19th century and for the first time its postulates were put together by Galton and triggered a series of measures in Western society that sought to improve the genetic inheritance, not only of the physiological characteristics of the population but also their intellectual and moral capacities. The means to achieve such improvement included marriage restrictions, selective sterilizations and control of immigration (Galton, 1904). Later, after the Second World War, these approaches were stigmatized after the Nuremberg trials and became banned, although not in practice in several countries (Tännsjö, 1998; Yap, 2007).

Thus, during the 80s of the last century, a second approach took place with a series of measures focused on preventing the spread of the disability–and disabled subjects–in society (Hens et al., 2013; Thompson, 2017). These measures included prenatal diagnosis, favored by the approval of abortion laws. Shortly after, techniques for preimplantation genetic diagnosis and screening, thanks to the development of assisted reproduction techniques, were largely implemented. All this prompted a new legislation that was progressively adapted to new demands of society (Fagot-Largeault, 1987). Simultaneously, dissenting voices appeared that criticized the commodification of human life, its conditioning and its designing (Hirschman, 1991). Contrary, other authors emerged criticizing the alarmism and encouraging the development of suitable legislation to avoid abuses (Resnik, 1994). Consequently, the role of independent, third party institutions become essential to evaluate the ethical dimension of the new techniques (Pergament and Bonnicksen, 1994) and fostered new regulations based on empirical data and not on moral abstractions (Bonnicksen, 1994). In this line, for example, the AMA Council on Ethical and Judicial Affairs put forward the criteria for accepting prenatal diagnosis and preimplantation genetic diagnosis, provided it was directed to therapeutic ends (AMA, 1994; Agar, 1995; Nicholson et al., 1995). Even so, the preventive selection of embryos, the manufacture of experimental embryos and the accumulation of cryopreserved embryos, led to new debates. As the therapeutic purpose became more diffuse or gave way to enhancement interventions, the debates shifted towards aspects such as the identity, benefit and dignity of the new creatures (Agar, 1995; Davis, 1995; Vines, 1995; Glannon, 1998).

A new phase was initiated in the 90s when novel cloning technology was developed and proposed for the acquisition or improvement of capabilities which are above the “normal” parameters for a human being (Richter and Bacchetta, 1998; Scully and Rehmann-Sutter, 2001; Greely, 2004). This new approach was called “enhancement” and included the acquisition of physiological, genetic, cognitive, and moral capabilities (Faust, 2008; Gordon-Solmon, 2015; DeGrazia, 2016). From the mid-1990s to the present, various initiatives have appeared trying to apply genetic enhancement to the individual for non-therapeutic purposes by means of manipulating, eliminating, or incorporating specific genes in adult subjects or in the germline (Blackburn, 2004). What makes this new phase in quite distinctive is the implication that the intervened individual will survive, will transmit his/her genes and will become a subject of rights. The appearance of Dolly, a cloned sheep in 1997, would start this new period including social alarm before the unknown and reactions calling for legislative caution. In 1997, both American and European legislation proposed a ban on human cloning (Newman, 2003; Hildt, 2016) which only had territorial influence. Despite these bans, there have been proposals for new therapies that take advantage of newer genetic technologies and which are loaded with controversy, such as human cloning involving nuclear transfer (Robertson, 1998), mitochondrial replacement (Rubenstein et al., 1995; Richter and Bacchetta, 1998), modification of the genomic map (Agar, 1995; MacKay et al., 1997), and genetic edition CRISP/Cas9 (Ishii, 2017a; Ishii, 2017b). Although the regulatory warnings seemed unanimous, the legislations were progressively adapted to the evolution of the different research evaluation committees. Therefore, there is a greater development of ethical objections and restrictions to research on severe medical conditions in some associations such as ESHG and ESHRE which remain more inclined to encourage social debate and to establish a moratorium (De Wert et al., 2018). However, other associations such as ASHG and NASM tend to raise the social and political problems involved in conducting this research (Lyon, 2017).

This new paradigm (“enhancement”) raises a recurrent question in the general health care field: what is the threshold between therapy and enhancement to intervene? Undoubtedly, the will to cure, intrinsic to the medical profession, is at its best in the effort to develop therapies targeting the cause of the problem, be it at the functional origin or at the structural level. In this context, gene therapy is a medical intervention which is considered proportionate in its intention and in its means — to recover lost health according to what it is to a human individual. On the contrary, genetic enhancement would try to modify non-pathological human traits and optimize capabilities in the individuals (NHGRI, 2018). Without a doubt there exists an intrinsic desire for the human zenith, constructed on a theoretical conception of what a perfect human being ought to be. And it is precisely this vision that is currently being challenged and questioned: the very concept of human identity, paired with succeeding questions: is that identity immutable? Are there arguments for not modifying it? And how far can it be modified? (Bostrom and Sandberg, 2009; Greenbaum, 2013; Macpherson and Segarra, 2017), and what are the long-term psychological and social consequences on individuals and populations (GüellPelayo, 2014; Cabrera, 2017; Ishii, 2017b)? In fact, all these issues frame the ethical challenges of “genetic enhancement” and, specifically, the genetic enhancement applied to the individual’s germline, aimed to improve the capabilities of the human subject.

These interventions are becoming an incipient new field of bioethics where the ELSI perspective is essential at its core (Henderson et al., 2012; Greenbaum, 2013; Macpherson and Segarra, 2017; Ishii, 2017a; Ishii, 2017b; De Melo-Martín, 2018; Tamir, 2018). This perspective permeates and affects all legislation of the clinical and pharmacology fields, subject to strict ethical controls, and associated to the *precautionary principle* (Gonzalvo-Cirac et al., 2013). This principle is applied in other fields too, such as protection of the environment (Rippe and Willemsen, 2018). In this context, a key question arises: is this sensitivity regarding the environment also present for genetic enhancement interventions in the individual’s germline? To answer this question, we carried out a literature review on the moral dimension of the problem and its non-clinical argumentation, that is, the argumentation generated in the process to assess the ethical dimensions to reject or support genetic enhancement carried out in the germline (Sparrow, 2014b; De Wert et al., 2018).

## Trends

There is an on-going debate on germline genetic enhancement within the public media and amongst specialists (Henderson et al., 2012) which manifests certain distrust towards solutions coming from the field of philosophy (Hayry, 2003; Coggon, 2011; Selgelid, 2014). This leads scientific debates to commonly end up on discussions around the political and ideological spheres with trends which oscillate between the so-called bioconservatives *versus* bioliberals factions (Roache and Savulescu, 2016). Both of them fluctuate between the anxiety before a new unpredictable technology and the demystification of their dangers (Cartier-Lacave et al., 2016). Even so, there is no shortage of attempts to reconcile both tendencies and seek a third way (Roduit et al., 2013; Shapshay, 2012; Qiu, 2016) that is able to integrate elements of both. For this reason, we have synthesized the reflections extracted from the various studies and grouped them into three main trends: preventive, proactive, and regulatory. It is worth noting that these tendencies show certain intertwining of their authors’ arguments and opinions but keeping their different starting points.

- *Preventive trend*. This trend groups diverse currents of thought whose common element is the attempt to preserve the human nature from the initiatives of germinal line modification. The studies in this group contain reflections from the Christian tradition (Polkinghorne, 2004; Massmann, 2018) and deontological philosophy (Jensen, 2011; Kim, 2017). They caution about the risk of modifying the essential element of human corporality, the DNA. The most representative author is Habermas (2003), who tries to analyze the reasons why we do not accept inherited nature and therefore would want to modify it genetically. It is an approach already evoked by other philosophers such as Kirkegard, Heidegger, or Jonas (Malmqvist, 2007; Christiansen, 2009), but Habermas goes a step further and argues that genetic enhancement in germline is to use the human being which may end up ‘making use’ of his being. From Habermas’s perspective, the preservation of the “non-chosen” or inherited nature, would protect us from ourselves (Neil, 2008), a protection that could be radically degraded if market laws were deployed in the reproductive industry (Fox, 2008). These laws would eventually determine the criteria for any action facing enhancement, including genetic doping (Gaffney and Parisotto, 2007; McKanna and Toriello, 2010), gene patents (Rodriguez, 2016; Du, 2018; Greenbaum, 2011), or competition between enhanced beings (Jensen, 2011). An additional critical approach may be added, fueled by the instability and insecurity of reprogenetic techniques leading to unpredictable consequences, in which case, use would be irresponsible (Fox, 2010; GüellPelayo, 2014; Hildt, 2016; Newman, 2017; Fox, 2018). This would be particularly relevant regarding the modification of the germline by genetic editing techniques (Niklas et al., 2015; Reagan, 2015; Sykora, 2015; Qiu, 2016). In summary, these studies may suggest that a balanced equation of risks and benefits would not suffice to determine the ethical assessment and morality of a technique. Rather, deeper understanding of key concepts identity, nature and dignity is necessary (Chan, 2015; De Melo-Martín, 2018; Jensen, 2018).
- *Proactive trend*. This trend rejects any intellectual barrier to research and claims to investigate freely, expanding knowledge, and eliminating alarmism (Harris, 2015). Therefore, they propose to overcome the precautionary principle and dismiss the arguments of the slippery slope in order to avoid slowing down the development of science (Pattinson, 2000; Bailey, 2001; Bernal, 2005). Thus, these authors propose cloning of germline DNA without any barriers (Robertson, 1998); the non-therapeutic purposes of preimplantation genetic diagnosis to select the individual’s features (Roberts, 2002; Sperling, 2011); the desire for greater intelligence by means of the selection of alleles (Kirk, 2003); or the selection of children according to their human potential (Gordon-Solmon, 2015), even if these initiatives were theoretical and utopian. It is common in these studies to denounce the impediments of moral restrictions (Smith et al., 2012; Murphy, 2014) and the cautions against cloning and genetic editing (Bernal, 2005; Fenton, 2006; Resnik and Vorhaus, 2006; Powell et al., 2012) although there is also moderate unwillingness to create hybrids between species (Robert and Baylis, 2003; Savulescu, 2003). In general, any opposition from philosophical, religious or political origin to scientific progress is questioned (Roberts, 2002; Brooke, 2004; Smolin, 2004) and a more empirical, less speculative analysis is pursued (Chyrowicz, 2001; Blackburn, 2004; Selgelid, 2014). These studies transmit the perception that there are social needs which are imperative to meet urgently and any attempt to hinder (*obstaculizar*) this process may be considered an attack, not only to progress, but also to social and global welfare.
- *Regulatory trend*. Another group of studies shows great sensitivity for the consequences, positive and negative, that may accompany genetic enhancement and try to solve it by proposing the need for clear legislation as a result of a public, open, and reflective debate (Marden and Nelkin, 2000). This trend tries to be an in-between solution between the positions of the two previous trends (Evitt et al., 2015; Thompson 2017). The regulatory trend anchors its main argument on the utilitarian pragmatism approach. Thus, it proposes decisions based on the function they may generate on the individual and in society. At the same time, this tendency considers essential to rely on strong laws to avoid abuses, keeping open to debate certain interventions, marked by individual or social need: e.g. cloning for sterile couples (Strong, 1998), the elimination of defective embryos (Verlinsky, 2005), the selection of embryos with antisocial genes (Tabery, 2009), the gamete planning (Delaney, 2011) or the experimentation with embryos without procreative purposes (De Miguel Beriain, 2019). At the same time, supporters of this regulatory approach also show concern for the social implications (Marden and Nelkin, 2000), especially injustice and inequality that could generate (Shapiro, 2005; Sparrow, 2015). There is, therefore, a great sensitivity to understand the consequences (Mehlman, 2003, Mehlman, 2005; Delaney, 2011; Anomaly, 2018) and a special interest to ensure coherent, global and coordinated legislation (Mackenzie, 2005; Ishii, 2014; Kaebnick, 2017; Kanaris, 2017; Lyon, 2017; Ishii, 2017a; Ishii, 2017b; Liao, 2019).

## Concepts

As indicated above, studies on genetic enhancement on the germline employ concepts which are used beyond the scientific–experimental dimensions of the problem and reach an ethical–logical dimension, more typical of philosophical approaches (Shapiro, 2005). This review has allowed us to highlight four significant concepts that are embedded in all debates and discussions addressing genetic enhancement: benefit, autonomy, identity, and dignity. Moreover, its significance differs in the various trends (**Table 1**) and a short-term consensus on its importance and relevance is not foreseen.

- *Benefit*. This is the focus of the discussion which configures the basis of the enhancement and is determined by the good that is pursued. For this reason, it is frequently addressed in most studies. The concept has been linked to the difference between the concepts “therapy” and “enhancement” which could also be interpreted as antagonists between the actions to “recover capacities” and means to “add capabilities” (Du, 2018; Thompson, 2017). Based on this divergence, a first reflection looks into the value of the arguments promoting genetic enhancement in germline (Neil, 2008) if the questions of what and why to enhance are not solved beforehand (Henrich, 2011). A second reflection comes out from the supposed benefit or harm caused through modification of the DNA, the genes or the human identity (Ebbesen and Jensen, 2006; Fenton, 2010). Some authors consider that modifications of human nature are advantageous (Powell et al., 2012), while others consider it detrimental to human beings, due to the potential corruption of the human genome (Sykora, 2015). In any case, the term benefit seems to be the most used amongst the authors and the foundation to carry out improvement and enhancement interventions.
- *Autonomy*. The subject’s autonomy is a condition present *sine qua non* in any human initiative and makes possible the subject’s informed consent. Thus, can reprogenetics cause detriment to individual autonomy? The answer seems very controversial (Mameli, 2007; Murphy, 2014; Schenker, 1997) due to deficient criteria with a comprehensive ethical assessment (Selgelid, 2014). However, since the implementation of embryo selection techniques by means of prenatal diagnosis and preimplantation genetic diagnosis, there is a perception that these interventions may alter the autonomy of manipulated individuals (Malmqvist, 2007; Henrich, 2011). This is especially evident when genetic enhancement is pursued: First, there are two wills which merge, the will of those responsible for the enhancement (e.g. parents, guardians or institutions) and the will of the enhanced individual (Tamir, 2018) which may not always coincide. Furthermore, a second dilemma is posed: the autonomy of parents who want to have a child may be deeply conditioned by the balance between their personal interests and the altruism of their action (Gordon-Solmon, 2015; DeGrazia, 2016; Jensen, 2018).
- *Identity*. In its classical conception, human identity is considered what characterizes the human individual. It is a concept that integrates the biological basis and the rational features of the human being. Often it is assimilated to the concept of Aristotelian nature, as described by Habermas (2003). However, a new definition and conceptualization of the meaning of identity seeks to include new technologies of genetic transformation (Shapiro, 2005) and other aspects such as autonomy, interpersonal relationships and longevity (Glannon and Harris, 2002). Nowadays, the lack of clear meaning and definition of the term identity is causing confusion in the debate on mitochondrial replacement and nuclear transfer techniques, as well as the proper identity of embryoids and chimaeras (Scott, 2017; De Miguel Beriain, 2019). In addition, the dilemmas of social identity of children generated *in vitro* including confused filiation which consequences have not yet been studied in depth (Rose and Novas, 2005; Lock and Nguyen, 2010) are added to the above problems. It is generally assumed that the concept of identity will be decisive to guide reflections on the moral status of the embryo (DeGrazia, 2012; Francis, 2015).
- *Dignity*. It is the most discussed concept in the debate on germline genetic enhancement and it is strongly contextualized and linked to the concept of identity (Savulescu, 2003). In spite of being used repeatedly, it is not well defined in the debate and it remains unclear (Henrich, 2011). Originally, this concept was mostly discussed in the debate on cloning (Caulfield, 2003), chimerization (Robert and Baylis, 2003; De Melo-Martín, 2008), and cryopreserved embryos (Glannon and Harris, 2002; Ehrich et al., 2010). Hence there have been repeated attempts to develop new conceptualizations rooted on neurological basis, consensual basis or rational basis (Bostrom and Sandberg, 2009; Jotterand, 2010; Chan, 2015). Undoubtedly, Habermas is the author who develops most deeply this term, dignity (p.29) (Habermas, 2003) in the field of genetics, especially due to its relationship with the concept of human nature. This concept would confer an infinite value to the fact of being human which would grow to be the foundation for an unconditional respect towards individuals and their human rights (Habermas, 2003; Christiansen, 2009).

**Table 1:** Summary of trends from the description of the main concepts.

Trend	Concepts			
	Benefit	Autonomy	Identity	Dignity
Preventive	The human good is the perfection of its being	Limited by the dignity of the individual	Determined by its nature, beyond its natural conditions	Based on its ontological nature
Proactive	The human good is the individual’s well-being (a good life)	Limited exclusively by the biological laws	Determined by their will, based on technological advances	Based on the development of its capabilities
Regulatory	The human good is the consensual social well-being	Limited by the laws that regulate society	Determined by social consensus after an open and regulated debate	Based on consensual legislation

## Current Controversies

Next, we deal with the main aspects that have caused greater controversy in scientific, technological, legal, or philosophical forums about the existence of a human genetic identity and the free initiative to modify that identity (Mehlman, 2003). This framework does not include other types of human improvement that science puts forward–physiological, cognitive or moral improvement through external elements such as drugs, surgery, or somatic genes–even if the focus remains similar: the happiness and well-being of individuals (Bostrom and Sandberg, 2009; Savulescu et al., 2011). The fundamental difference of these tendencies with genetic enhancement in germline lies in a concept previously mentioned: the autonomy of the individual. The recipients of the genetic enhancement have not chosen to be better, something that is required for any other pharmacological, neuronal, or surgical improvement, which usually includes free, informed consent. Therefore, we focus on specific areas in which genetic improvement affects the fundamental rights (including future identity, dignity, and good lifetime) of individuals which are especially vulnerable and without autonomy, such as embryos or a newborn (Liao, 2019).

### Procreative Beneficence

Faced with the technology available and the possibility of predictably beneficial enhancements, a question comes out unstoppably: should not the selection of genes be mandatory? Shouldn’t governments be allowed to promote or prevent certain genes upon citizens in a similar way as it is done with vaccination programs (Kanaris, 2017; So et al., 2017)? Anticipating these issues, Savulescu developed what would be called “the Principle of Procreative Beneficence” by which parents would be morally obliged to discard an embryo with potential criminal genes and at the same time choose the embryos that have the most favorable genes for himself and for society (Savulescu, 2001; Savulescu et al., 2006). This issue was already an old controversy, which was raised even before the emergence of prenatal diagnostic techniques and preimplantation genetic diagnosis: the obligation of the principle of beneficence against the foreseeable diseases present in the embryo, e.g. Huntington disease, Asperger syndrome, Down syndrome, cancer, cystic fibrosis, and spina bifida amongst others (Harris, 2006; Walsh, 2010; Bosslet, 2011), removing the procreative autonomy of the parents (Faust, 2008; DeGrazia, 2016). This state-driven intervention approach to eliminate the supposed “antisocial” embryos has been strongly criticized (Tabery, 2009; Bosslet, 2011) due to the fact that it would require a substantial drift in the defense of human rights: it would not differentiate between a desire for moral enhancement and a mandatory action to implement it (Saunders, 2015). The root of this disruption may be found in the moral imperative–the moral good as obligation–and its mandatory application leading to morally designed individuals (Holland, 2016). In the same line, psychological pressure on parents would be especially significant (Bonte et al., 2014) to encourage them to choose the “best” embryo amongst several, that would be the most “valuable,” “intelligent” or “excellent,” a quantification attitude that would seem incoherent, or at least surprising, for parents with unconditional affection for any of their children (Tonkens, 2011; Jensen, 2018). Therefore, some authors have taken another approach and are inclined to transform the obligation into suggestion, option or advice (Jacobs, 2015; Carter, 2015; Francis, 2015; Sparrow, 2015; Kanaris, 2017; Liao, 2019).

### The Value of the Disability

The principle of procreative beneficence raises a new question: who is entitled to decide what an advantage is (Karpin, 2007; Macpherson and Segarra, 2017)? It is not clear whether some enhancements are desirable *per se* or whether a disability is deprived of any value (Nunes, 2006; Francis, 2015). According to the *expressivist objection* current, the elimination of disabilities would be a loss of human identity for those who suffer them (Alper et al., 2002; Malek, 2010; Collins et al. 2016; Shakespeare et al., 2017). Within the same context, then, we consider how negative interventions should be assessed: the selection of features that we may consider detrimental for the future child, e.g. deafness or Asperger, may also present some kind of benefit (Karpin, 2007; Walsh, 2010; Graber, 2017). Would there be an obligation to avoid them? At this point, some authors have raised the concept of the “asymmetry of the damage,” by which the benefit of living with a disability would not compensate the damage of having it (Glannon and Harris, 2002; Sparrow, 2012; Francis, 2015). Furthermore, the anti-equality shadow appears simultaneously, either by genetic enhancement or by embryonic selection technologies, since their application effectively would impose social segregation between the enhanced individuals and the non-enhanced subjects, who would identify themselves with the disabled (Cavaliere, 2018). It is here where the legal regulatory bodies need to deepen the knowledge and the consequences of their implementation since mandatory actions to eradicate disability could lead to the extermination of full groups (Kanaris, 2017; Thompson, 2017; Ishii, 2017a).

### Gender Selection

Undoubtedly, sex selection for non-therapeutic reasons has already generated a multitude of controversies (Arnold et al., 2002; Sperling, 2011; Winckler, 2002). Currently, a new debate is taking place regarding the advantage or disadvantage of sexual dimorphism (Sparrow, 2010a) focused on the supposed “normality” of the existence of two sexes and whether one should prevail over the other (Kahane and Savulescu, 2010; Slatman et al., 2010; Sparrow, 2010b; Sparrow, 2012). If that normality were to be questioned, would it be justifiable to eliminate sexual dimorphism and select the best female or male genes to design an asexual being (Kahane and Savulescu, 2010)? This proposal could even go as far as to suggest the mandatory choice of the female subject by the tutors or the reproductive leaders upon consideration and assumption that the most aggressive genes would come from the male individual, an extremely controversial trait (Slatman et al., 2010; Sparrow, 2012; Casal, 2013). According to other authors, this debate is insubstantial in nature because sexual dimorphism has a neutral effect on human development (Kahane and Savulescu, 2010) and the normality of each of the sexes is accepted (Sparrow, 2012). In spite of everything, the appearance of ectogenetic technologies will possibly repeat the controversies generated when the embryo selection techniques were and currently are applied to sex selection (Kendal, 2017).

### Creation of Chimeras, Hybrids, and Embryoids

Their creation has always raised rejection. The recent change of social mindset has been preceded by their potential usefulness in research, primarily related to therapeutics (Brickman and Serup, 2017). This approach was fueled when IVF surplus human embryos began to be used to produce stem cells, thus justifying the elimination of any restriction on embryonic experimentation (Robert and Baylis, 2003; Savulescu, 2003; Ehrich et al., 2010; Volarevic et al., 2018; De Miguel Beriain, 2019). In spite of everything, the doubt and aversion have persisted due to the uncertainty of the moral status of the hybrid human–animal individual (Streiffer, 2005; Kaebnick, 2017). Moreover, this uncertainty acquires special relevance when the human–animal interaction may affect the brain structure and functionality as well as the gametes (Dolgin, 2016; Levine and Grabel, 2017). The current trends range from the elimination of any restriction due to lack of ethical reasons, to the concern for individual and societal consequences (Palacios-González, 2015; Hyun, 2016; Rodriguez, 2016). We believe that the solution can only be developed departing from a clear conceptualization of their moral status, a term still not agreed upon for embryos and much less for chimeras (Giacomini et al., 2007; De Melo-Martín, 2008; Eberl and Ballard, 2009; Chan, 2015; Munsie et al., 2017; Hübner, 2018).

### Genetic Untying

The problem of genetic untying or the absence of any genetic linkage between the embryo and the parents surged with the requirement of anonymity of the donors of the gametes for *in vitro* fertilization and surrogate motherhood. Further speculation to this debate has been added when taking into account the dissociating effects of new technologies, such as the IVR technique (*in vitro* iterated reproduction) where the gametes are generated directly from the embryos and mitochondrial replacement technologies that involves genetic material from three gametes (Bredenoord et al., 2011). In this technique, the intervention is not carried out directly on the embryo but on its precursors, e.g. its gametes (Delaney, 2011). In all these cases, the result is an embryo genetically disconnected from its progenitors either because the gametes used are generated *in vitro* (Palacios-González et al., 2014), or they are derived from multiple gametes, or they have been generated by iterated reproduction (Sparrow, 2014a). The end result is a “genetic orphan,” an individual without living parents, since the most immediate ancestor would be deceased embryos (Sparrow, 2014b); a situation which cannot be assimilated to the natural generation of monozygotic twins (Douglas, 2014). The concern caused by mitochondrial donations and transfer techniques is flagrant generating new legislative doubts about the conditions for its application (Ishii, 2014; Harris, 2015), especially regarding anonymity (Brandt, 2016). Thus, there is discussion on the emergency of a new idea within the human procreation context: the abolition of procreative filiation and the possibility of raising design individuals without kinship (Palacios-González et al., 2014; Roache, 2016).

### Psychosocial Inequality

The alteration of the social balance has always been a great concern for the agents involved in enhancement efforts (Davis, 1997; Davis, 2009). This has been even more explicit if it could affect the future of the child (Sparrow, 2016; Krutzinna, 2017). The lack of restrictions makes it possible to foresee the social drift that these technologies may cause (Mehlman, 2005; Ishii, 2017b) although some authors consider this caution for inequality disproportionate (Fenton, 2010) and limit their scope to assess of damage that might be predicted after implementation of enhancement interventions (Persson, 2012). Even so, the fear of a social disadvantage in unenhanced children is recurrent and considered immoral and unjustified due to its potential for discrimination (Jensen, 2011), the possible unknown consequences in complex thinking and reasoning (Rosoff, 2011) and other long term secondary effects (Glick, 2011). In this sense, the alarm over the application of techniques that we do not understand completely or do not know how to control (GüellPelayo, 2014), ranges from conditioning and alteration of the child’s future (Bredenoord et al., 2014) to a series of enhancements that eventually may lead to social fragmentation and disintegration (Sparrow, 2015). For the moment, the debate has crystallized in the production of legislation that continues to be diffuse or even contradictory (De Souza, 2015; Kim, 2017; Tamir, 2018).

## Future Developments

Germline genetic enhancement presents important challenges that should be progressively clarified, mainly to prevent reproductive technology from being hijacked by its own advances. We must bear in mind that the ultimate reason for these initiatives is a concept of elementary ethics: the common good and the happiness of individuals. In essence, this is the engine that activates actions that promote, limit or regulate genetic technologies progress. We understand that a thorough argumentation within the ELSI framework, including the reprogenetic arena (NHGRI, 2018), would be a way to ensure a proper application and implementation of any genetic enhancement initiative, whether theoretical or practical. In view of the studies reviewed, this working framework would have an impact on three fundamental questions where the ethical implications of the new technologies are rooted:

*a) The moral status of the embryo*. The issue was initially raised when the technologies that affected the integrity of the embryo were implemented pushing the debate about its nature and identity (Fox, 2010; DeGrazia, 2012). The discussion and social debate about the embryo’s moral status became especially relevant when it came to specific issues such as the fate of hundreds of thousands of cryopreserved embryos, their use for experimentation, the consensual arbitrary limitation 14 days for research and the patented embryos (Ehrich et al., 2010; Cavaliere, 2017; Chan, 2017; Appleby and Bredenoord, 2018). In fact, the fragility of this status is what allows the manipulation of embryos through genetic editing, provided that they are subsequently eliminated and not used in the germline. (De Miguel Beriain, 2019). More recently, research the creation of transformed clones, hybrids, chimeras, and embryoid bodies has added a greater uncertainty to a question that remains unclear. It is not known exactly what the entity of embryoids, organoids, or synthetic embryos may be (Chan, 2017; Kaebnick, 2017; Munsie et al., 2017). Society is looking for answers to formulate the most elementary question: what is it? or who is it? The reflection on the most basic definition of the principle of non-contradiction, something cannot be both true and not true in the same way and at the same time, pushes the debate towards a binary solution. The possibility that there may even be allowed the slightest manipulation of embryo beings, predictably human, without a defined identity status and without any biological linkage with anyone, could lead to a profuse and disturbing legislation to coax their adult development, whether they be clones, chimeras, enhanced chimeras, or enhanced embryos (Mason, 2017; Scott, 2017; De Melo-Martin, 2018). In this context, the framework of reflection goes beyond the purely scientific or technological sphere and invades the anthropological arena. Thus, the philosophical framework in it anthropological dimension would be the one qualified to define the ontology of beings whose status is, apparently, diffuse, as well as their moral, social and legal identity (Eberl and Ballard, 2009; Qiu, 2016). We consider it essential to determine the ontological status of these beings because its clarification will serve as a starting point to determine the administration of their destiny, either as non-human, their use, their property, or as human being, their respect and their rights (Polkinghorne, 2004; Streiffer, 2005).
*b) The rights of the modified individual*. The question of genetic enhancement on the germline would not imply special obstacles if it were not because it breaks into the most intimate sphere of human beings in two dimensions: first, the decisions that are made and implemented will be irreversible and second, the genetic characteristics are likely not to have been chosen by the individual itself but imposed on him/her, not by nature but by a particular will. This situation was, precisely, the scenario that was aimed to avoid: the imposition by “nature” on the individual of certain characteristics that he or she had not chosen but rather had to accept resignedly. Would we be falling again into an injustice? What should be done if the modified individual does not accept his modified status? It could be argued that it is a scenario similar to an individual conceived in a natural way, (e.g. through sexual intercourse) who would not accept his/her condition or a specific feature (e.g. height, intelligence or even an inherited pathology, etc). However, there is an essential difference between both scenarios. The natural inheritance cannot be imputed to anyone, a key element for legislative purposes since the individual is not responsible for it. However, modified inheritance is the result of the deliberate action of other individuals (e.g. parents or guardians) who decide how the child should be. Therefore, they are fully responsible for the action carried out in the child with legal responsibility (Sundby et al., 2018). In fact, there is general consensus about the non-implementation on children interventions with serious risks which may be not properly balanced with the benefit received from it (Delaney, 2011; Powell and Buchanan, 2011). It is worth noting that therapies applied to children, who cannot agree to an informed consent (e.g. vaccination, corrective surgeries, etc) are only ethically justified when the benefit is the survival or the integrity of the newborn or child. In addition, those therapies should always respect their identity and should not lead to the modification or selective destruction of other individuals (Millum, 2014; Hendrix et al., 2016).

But the existence of enhanced genes in the newborn inflicts characteristics that make that individual unique to himself and others (Shapiro, 2005). Here a set of questions come out: did he have the right not to be enhanced? Did he have the right to reject imposed selection or imposed genes, positive or negative, not by the laws of nature but by the will of his managers? Does he have the right to a biological filiationties (Malmqvist, 2007; Karpin, 2007; De Melo-Martín, 2018)? If these rights existed, they would generate a dilemma of difficult solution: the need for an informed consent to undergo the enhancement procedure and at the same time the right of refusal to be modified (Rodriguez, 2016). Furthermore, it would be unclear how to resolve its true biological identity–the cause and reason for its origin–and its right to biological filiation, a question that has already led to the modification of legislation about the anonymity of the donor of the gametes. In brief, will the enhanced individual have the right to know his detailed origin? Or the purpose of the enhancement carried out in him (Reagan, 2015; Sykora, 2015)? The analysis of the right to be a genetically enhanced individual is still in its infancy because it has yet materialized. In fact, some authors speculate that this right would depend on external factors to the individual (Tamir, 2018), an approach that may cause additional restlessness. Consequently, we consider it essential to exercise extreme caution in all interventions (Holm, 2019), in order to generate a truly human technology (Nordberg et al., 2018).

*c) The ethical, social and legal responsibility of the enhancement agents*. The technologies of germline genetic enhancement carried out on individuals raise additional questions regarding the ownership of the action: Who is accountable for the changes, positive or negative, executed in individuals (Rodriguez, 2016; Du, 2018)? There are a variety of agents that could be expected to be accountable: the parents, the tutors, the researchers, the corporations, or even the State (Sparrow, 2016), that is, any agent interested in the realization of the enhancement (Millum, 2014; Kanaris, 2017). This accountability seems similar to the responsibility that is acquired when a therapeutic process is applied, however the motive and objective of the intervention produces a key difference between one and the other. In the case of therapies, it is understood that the responsibility is universal because it responds to an intrinsic need of every human being: the ultimate goal of the intervention is the health improvement of the intervened individual (Bonte et al., 2014). Meanwhile, in genetic enhancement interventions, these needs are exceeded: the enhancement pursues to satisfy the desire of some individuals who decide the type of enhancement without clarifying who will assume the consequences (Thompson, 2017; Ishii, 2017a; Anomaly, 2018). Although some entities (NHGRI, ASHG, and ESHG) have expanded their scope of studies and include risk assessment, we agree with the authors who consider that these measures are insufficient (Mehlman, 2009). Given that germline genetic enhancement application occurs at the beginning of the living stages of the human being (embryos, newborns, or even children), it is especially important to assess the long-term psychological and social consequences, with the conviction that there are red lines that should not be crossed (Mehlman, 2005; Mehlman, 2009; Greenbaum, 2013).

## Conclusion

It is probable that the desire for specific human enhancement will become a reality and, consequently, some agents will implement the germline’s genetic enhancement in society. For this reason, we consider essential to create effective expert panels and committees with society’s feedback (Kaebnick, 2017) that could elaborate global normative documents, rooted and established on universal ethical principles (Ishii, 2014; Lyon, 2017; De Wert et al., 2018).

The information about the creation of enhanced twins in China (Regalado, 2018) reinforces our conviction about the need to put forward the underlying reasons that support future legislation aimed at prohibiting or allowing enhancements. Otherwise, if the different reasons are circumstantial without a deep foundation, the laws will be ineffective.

We propose the precautionary principle as a means to navigate ethics’ uncertainties and as the point of departure to assess moral enhancement. Certainly, an abusive application of the precautionary principle would lead to its ineffectiveness. Conversely, that precautionary attitude may improve the objectives and the means regardless whether it is directed to protect the autonomy of adults, the global human welfare or the dignity of the individual. We think that these concepts may structure and configure any advance in germline genetic enhancement technologies.

## Author contributions

IM and IS developed and conceptualized the initial idea and carried out the revision process; MVR, IM and IS contributed to the discussion and elaboration of the sections.

## Conflict of Interest Statement

The authors declare that the research was conducted in the absence of any commercial or financial relationships that could be construed as a potential conflict of interest.
